# Rhamnogalacturonan-I forms mucilage: behind its simplicity, a cutting-edge organization

**DOI:** 10.1093/jxb/erac094

**Published:** 2022-06-01

**Authors:** Susana Saez-Aguayo, Asier Largo-Gosens

**Affiliations:** Centro de Biotecnología Vegetal, Laboratorio Mucilab, Facultad de Ciencias de la Vida, Universidad Andrés Bello, Santiago 8370146, Chile; Área de Fisiología Vegetal, Departamento de Ingenería y Ciencias Agrarias, Universidad de León, E-24071, León, Spain

**Keywords:** Pectin biosynthesis, rhamnogalacturonan-I, seed mucilage, transcription factors

## Abstract

**Zhang Y, Yin Q, Qin W, Gao H, Du J, Chen J, Li H, Zhou G, Wu H, Wu A-M**. 2022. The Class II KNOX family members *KNAT3* and *KNAT7* redundantly participate in Arabidopsis seed coat mucilage biosynthesis. Journal of Experimental Botany 73, 3477–3495.

**Arabidopsis mucilage has been used for more than two decades to investigate and characterize different factors of pectin synthesis and modification. Using cytological, transcriptomic, chemical, and molecular approaches, Zhang *et al.* (2022) clarify the redundant role of KNAT3 and KNAT7, two KNOX Class II transcription factors, in mucilage rhamnogalacturonan-I (RG-I) biosynthesis through the regulation of a group of genes which coordinate the formation of these specific pectic domains.**

Seed mucilage is a hydrated gel-like structure produced by seed coat tegument of myxospermous seeds and participates in seed dispersion, seed germination, and the maintenance of the earth rhizosphere (Macquet *et al.*, 2007a; Saez-Aguayo *et al.*, 2014; Voiniciuc *et al.*, 2015a). The Arabidopsis seed coat mucilage has been used for >20 years to elucidate the roles of genes in the extremely complex process of cell wall synthesis and remodeling (reviewed in Šola *et al.*, 2018). Arabidopsis mucilage is mainly composed of unbranched rhamnogalacturonan-I (RG-I) and homogalacturonan (HG) pectin domains (Box 1) (Macquet *et al.*, 2007a; Voiniciuc *et al.*, 2015a). Both domains are enriched in the acidic sugar galacturonic acid (GalA), although these polysaccharides have quite different structures (Mohnen *et al.*, 2008). Indeed, in the RG-I backbone, GalA is alternated with rhamnose (Rha), while in the HG domain, the backbone is formed exclusively by GalA residues that can be methylesterified in their carboxyl groups (Mohnen *et al.*, 2008). Mucilage polysaccharides are deposited in specialized seed epidermal mucilage secretory cells (MSCs) throughout seed development. The active deposition of mucilage polysaccharides led to the formation of a central column called the columella in a highly regulated process (Box 1A; Western *et al*, 2004; Macquet *et al.*, 2007a; Voiniciuc *et al.*, 2015b; Golz *et al.*, 2018). In their study, Zhang *et al.* (2022) provide evidence of the participation of KNAT3 and KNAT7 in this process by an exhaustive analysis of the mucilage phenotype of *knat3* and *knat7* single mutants and the *knat3knat7* double mutant using cytological, immunological, and biochemical approaches. The authors determine that KNAT7 and KNAT3 mutation affects seed epidermal cell morphology and observed a flattened columella in *knat3knat7* double mutants. Also, the authors determined less RG-I production and changes in HG structure which could explain the MSC phenotype.

**Box 1. Mucilage formation, composition, and structure in Arabidopsis seed coat epidermal cells.**

(A) Mucilage formation and deposition during seed coat epidermal cell differentiation.

Schematic representation of seed coat epidermal cell differentiation in Arabidopsis. At 6 days after pollination (DAP), previously formed starch granules (sg) are surrounded by a large vacuole (v) which pushes the cytoplasm (cy) to the cell periphery. Between 8 and 10 DAP, mucilage production is at its maximum, and polysaccharides are synthesized and deposited in the corner of the apoplast of epidermal cells. These polarized deposits force the cytoplasm into a central column filled with starch granules of mucilage polysaccharides. From 12 DAP, mucilage deposition has ended, and starch granules are replaced with wall material that forms a structure called the columella. In the epidermal cells, the central columella is surrounded by an enriched pectin pocket. C, columella; M, mucilage; dw, distal wall; rw, radial wall (Macquet *et al.*, 2007a; Voiniciuc *et al.*, 2015a; Šola *et al.*, 2018; Saez-Aguayo *et al.*, 2021)

(B) Detailed mucilage RG-I composition and structure described in adherent (AM) and soluble (SM) mucilage layers.

Mucilage is mainly constituted of RG-I and HG which are Rha- and GalA-enriched pectin domains. It also contains traces of cellulose, mannose, heteroxylan, and xylan polysaccharides, as well as galactose and arabinose contained in galactan and arabinan RG-I side chains. Evidence shows that adherent (AM) and soluble mucilage (SM) differ in composition. The AM structure contains RG-I, HG, cellulose, xylan, heteroxylan, and mannan domains, while the SM layer contains mainly RG-I, HG, and xylan. RG-I is subject to structural changes during mucilage maturation, and the final structure is different in SM and AM. It was determined that both mucilage layers are mainly composed of smooth RG-I with few galactan, arabinan, and xylan chains, which are more frequent in AM. In SM, the smooth RG-I form is more frequently detected at mature stages. In fact, RG-I from SM is produced with side chains at the immature stage which are removed during maturation. Recently, it was determined that mature smooth RG-I from SM can self-assemble into a multichain structure similar to microfibrils (plethora).

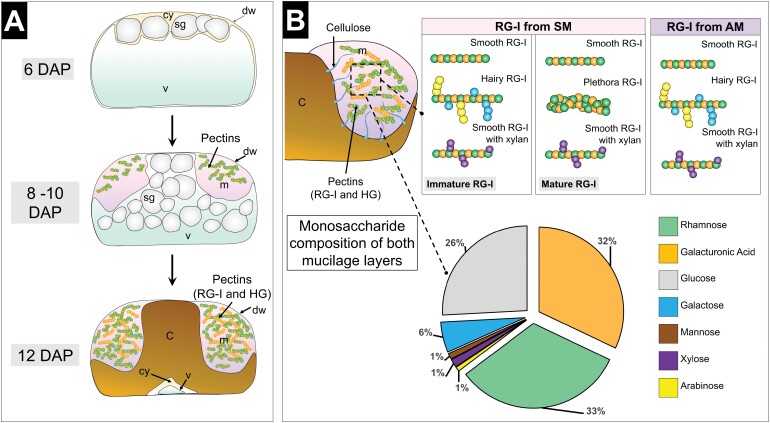

## Elucidation of the complex regulation of mucilage RG-I synthesis and structure

Thanks to the great work realized by Zhang and collaborators, the tangled ‘ball of wool’ which represents the regulation of pectin synthesis is a little bit clearer now. At the mature stage, mucilage has been characterized as a plethora of unbranched RG-I polymers with an average size of 600 kDa; therefore, it is formed by ~1800 units of Rha–GalA disaccharide (Box 2A; Macquet *et al.*, 2007a; Williams *et al.*, 2020; Saez-Aguayo *et al.*, 2021). Elongation of this ‘giant’ polymer is initiated by the coordinated action of enzymes localized in the Golgi apparatus and in the cytosol (Fabrissin *et al.*, 2019; Saez-Aguayo *et al.*, 2021). MUCILAGE-MODIFIED-4 (MUM4) converts UDP-Glc to UDP-Rha in the cytosol before the transport of the latter to the Golgi lumen by URGT2/4/6 transporters (Western *et al.*, 2004; Saez-Aguayo *et al.*, 2021). UDP-GlcA is transported from the cytosol to the Golgi apparatus by UUAT1 and, presumably, UUAT3, and is converted to UDP-GalA by glucuronate epimerase (GAE) (Box 2A) in the lumen of this compartment (Saez-Aguayo *et al.*, 2017, 2021; Parra-Rojas *et al.*, 2019). When substrates are in the lumen of the Golgi, they are incorporated into the nascent RG-I polymer thanks to RRT1 (rhamnosyl transferase 1), GATL5, GAUT11, and MUCI70 (galacturunosyl transferases), up to the formation of RG-I chains (Saez-Aguayo *et al.*, 2017, 2021; Takenaka *et al.*, 2018; Voiniciuc *et al.*, 2018; Fabrissin *et al.*, 2019). To date, there are no GTs described acting on RG-I ramification (arabinans and galactans). Recently, a hypothetical xylan side chain on mucilage RG-I has been proposed based on the evidence that xylan participates in mucilage adherence by the attachment of RG-I to cellulose. Xylan synthesis is highly coordinated with RG-I production, suggesting the existence of xylan side chains covalently linked to RG-I (Tan *et al.*, 2013; Voiniciuc *et al.*, 2015b; Ralet *et al.*, 2016; Fabrissin *et al.*, 2019; Saez-Aguayo *et al.*, 2021). However, the exact xylan association with the RG-I structure in mucilage still remains unclear, although MUM5 and IRX14 have been characterized as xylosyl transferases adding xylose into xylan structures in mucilage polysaccharides (Voiniciuc *et al.*, 2015b; Fabrissin *et al.*, 2019). During RG-I maturation, arabinan and galactan side chains are removed by MUM2 and BXL1 (Macquet *et al.*, 2007b; Williams *et al.*, 2020). No evidence clearly explains this strong change of structure but, considering that the presence of galactans and arabinans in the RG-I structure reduces the hydration ability of mucilage (Dean *et al.*, 2007; Arsovski *et al.*, 2009; Rautengarten *et al.*, 2011), it seems that the synthesis of RG-I side chains is required for compaction and organization into the dehydrated mucilage pocket, and their degradation would ensure the hydration and liberation of mucilage in the presence of water.

**Box 2. Current description of key factors acting on RG-I synthesis and structure.**

(A) Schematic representation of mucilage RG-I and xylan synthesis in Arabidopsis

UUAT1/3, URGT2/4/6, RRT1, GATL5, GAUT11, MUCI70, MUM5, and IRX14 have been implicated in the synthesis of mucilage RG-I and xylan molecules. URGTs and UUATs are proteins that ensure the transport of UDP-Rha and UDP-GlcA—the latter of which is transformed into UDP-GalA, the precursor of RG-I, by a glucuronate epimerase (GAE)—from the cytosol to the Golgi. The coordinated action of the two glycosyltransferases RRT1, a rhamnosyltransferase, and GAUT11, a galactouronosyl-transferase, build up the mostly unbranched RG-I backbone. As the RG-I is synthesized, the putative xylosyltransferase MUM5 adds a xylose that will constitute a xylan side chain by the addition of more xyloses by IRX14. The participation of GALT5 and MUCI70 in RG-I and HG synthesis was demonstrated, but to a minor extent. To date, no GTs involved in RG-I ramifications have been described. Once pectins are secreted into the apoplast, they mature by the removal of lateral chain ramifications realized by MUM2 and BXL1 which are a galactanase and arabinase, respectively. At the mature stage, RG-I has been characterized to be a plethora of RG-I molecules with an average size of 600 kDa, and is thus formed by ~1800 units of Rha–GalA disaccharide.

(B) Schematic model of transcriptomic regulation of genes implicated in mucilage RG-I production in seed coat epidermal cells

Fourteen transcription factors (TFs) have been reported to regulate gene expression involved particularly in RG-I formation and structure (Huang *et al.*, 2011; Ezquer *et al.*, 2016; Saez-Aguayo *et al.*, 2017, 2021; Golz *et al.*, 2018; Xu *et al.*, 2022). The complex formed by the TF TTG1–TT8–MYB5 regulates the action of TTG2 and GL2, both activators of MUM4, URGT2, URGT4, and GATL5, involved in RG-I synthesis. MUM1 has its own pathway, regulated by STK, and inhibits UUAT1 and URGT6 which have a discrete role in RG-I formation. Additionally, MUM1 is an activator of enzymes such as MUM2 and BXL1which remove RG-I branching. The TF DE1 BINDING FACTOR (DF1) is able to bind to GL2 and thus control MUM4 and GATL5. It was also described that TTG2 could control DF1 and GL2 expression, and DF1 is also able to repress the expression of TTG2 forming a loop of regulation. Finally, the complex described in this work demonstrates that MYB75–TT8–TTG1–KNAT3/7 activate MUM4, involved in RG-I synthesis in mucilage, and, at least, KNAT7 controlled the expression of IRX14, and MUM5 which synthesizes xylan ramifications. Regulations characterized in this study are shown with red arrows.

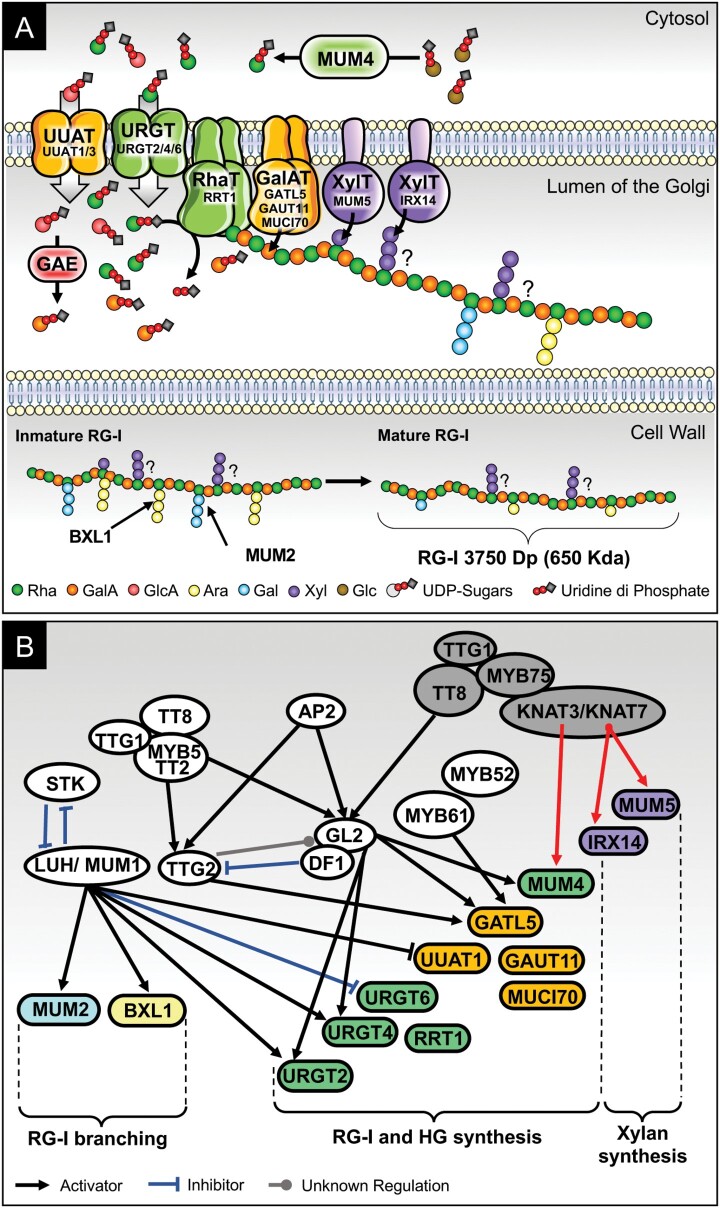

## KNAT3 and KNAT7 orchestrate genes involved in mucilage RG-I synthesis

As explained before, KNAT3 and KNAT7 are two transcription factors (TFs) that belong to Class II of the KNOX TF family and they are expressed in the seed tegument during seed development. Zhang *et al.* (2022) demonstrate that the lower expression of these TFs led to low accumulation of mucilage and the disruption of the formation of the columella, a phenotype which was particularly detected in *knat3knat7* double mutants. Considering that these mucilage defects have previously been observed in mutants of other TFs which regulate genes involved in RG-I synthesis, such as APETALA (AP2), TRANSPARENT TESTA GLABRA1 (TTG1), and GLABRA2 (GL2) (Golz *et al.*, 2018; Box 2), the authors expected that KNAT3 and KNAT7 also regulate genes involved in this process. To investigate this, Zhang *et al.* (2022) carried out a transcriptomic analysis from siliques of single and double mutants during four stages of seed development to get an idea about the genetic changes caused by the absence of *KNAT3* and *KNAT7*. They observed considerable changes in gene expression in single and double mutants related to numerous physiological processes. One of the most interesting changes is the reduction of expression of a high number of GTs and PMEs in the *knat3knat7* double mutant which are related to RG-I and HG synthesis and modification. To confirm this, the authors showed the reduction of expression of mucilage-specific genes, such as *MUM4*, *MUM2*, and *GALT5* which encode proteins focused on RG-I elongation and modification (Western *et al.*, 2004; Dean *et al.*, 2007; Macquet *et al.*, 2007b; Kong *et al.*, 2013) at early seed developmental stages, suggesting that KNAT3 and KNAT7 regulate this process. In a similar way, *knat3* and *knat7* mutation reduces the expression of *MUM2* in early stages of seed development. *MUM2* encodes a β-galactosidase that cuts the galactan side chains of RG-I detected in mucilage, confirming that both TFs act in synergy to regulate seed mucilage RG-I production and structure.

## KNAT3 and KNAT7 regulate mucilage RG-I synthesis by the activation of *MUM4* expression

Considering all the mucilage-related genes repressed in the *knat3knat7* double mutant, the authors explored whether these TFs could act as direct activators of any of these genes. By an elegant transactivation assay using a dual-luciferase reporter, the authors demonstrate that KNAT3 and KNAT7 act as redundant activators of *MUM4*, suggesting the existence of other activators for *MUM2* and *GATL5.* As it was previously described that KNAT7 can physically interact with MYB75 (PAP1) to regulate secondary cell wall formation in the Arabidopsis seed coat (Bhargava *et al.*, 2013), the authors suggest that the activation of those mucilage-related genes could be due to the formation of the complex TTG1–TT8–MYB75–KNAT7/3 mucilage MBW module. The mucilage MBW module can activate the expression of the TFs GL2 and TTG2, which in turn could modulate the expression of MUM4, GATL5, URGT2, URGT4, and other RG-I related genes (Western *et al.*, 2001; Kong *et al.*, 2013; Golz *et al.*, 2018; Saez-Aguayo *et al.*, 2021). Thus, the KNAT complex demonstrates a specific regulation of RG-I synthesis and modification, in parallel with other TFs already reported to regulate the expression of mucilage-specific genes described in detail in the Box 2B (Huang *et al.*, 2011; Ezquer *et al.*, 2016; Saez-Aguayo *et al.*, 2017, 2021; Golz *et al.*, 2018; Xu *et al.*, 2022).

## Step forward with seed mucilage from different species

Thanks to two decades of research, we have now made great advances in knowledge of the key enzymes and TFs in the regulation of synthesis and modification of the major mucilage component (RG-I). However, there is a lot of work still to do to understand the synthesis, modification, and, especially, the regulation of other minor mucilage components, such as HG, cellulose, arabinans, galactans, and galactoglucomannans, among others. However, as the research in this specific field advances, maybe we can wonder if all this knowledge of how the machinery works in the synthesis of plant polymers is relevant for the future. Recent research revealed the great potential of several pectic domains for human health (Ndeh and Gilbert, 2018). Indeed, during digestion, the colon is the place where intestinal microorganisms act as decomposers of polysaccharides, providing nutrients for probiotic bacteria and influencing the gut microbiome (Ndeh and Gilbert, 2018). The degradation of polysaccharides generates certain oligosaccharides that have known anti-inflammatory activity and boost the human immunity system, helping humans to stay healthy (Thomson *et al.*, 2018; Barbosa and de Carvalho Junior, 2021). Among the different structural domains of pectins, the HG domain seems to be the reactive domain for alleviating acute inflammation. Moreover, RG-I has promising effects for the treatment of different chronic inflammatory diseases such as periodontitis, rheumatoid arthritis, and also as a novel anti-ulcer agent (Nascimiento *et al.*, 2017). The question that is raised is: are we able to modify the pectin production system to obtain more reactive pectin with a greater effect on human health? Also, are we able to transfer this knowledge to crops such as flax or chia, to produce functional foods able to boost human health? These are some of the challenges that we could consider thanks to the research carried out on mucilage to date.
