# Correction to: Rhamnogalacturonan-I forms mucilage: behind its simplicity, a cutting-edge organization

**DOI:** 10.1093/jxb/erac296

**Published:** 2022-07-13

This is a correction to: Susana Saez-Aguayo, Asier Largo-Gosens, Rhamnogalacturonan-I forms mucilage: behind its simplicity, a cutting-edge organization, *Journal of Experimental Botany*, Volume 73, Issue 11, 2 June 2022, Pages 3299–3303, https://doi.org/10.1093/jxb/erac094

In the originally published version of this manuscript, figures were erroneously omitted from both Box 1 and Box 2. This error has now been corrected.

